# Microstructural Characterization of Melt Extracted High-Nb-Containing TiAl-Based Fiber

**DOI:** 10.3390/ma10020195

**Published:** 2017-02-17

**Authors:** Shuzhi Zhang, Shuling Zhang, Yanfei Chen, Jianchao Han, Changjiang Zhang, Xiaopeng Wang, Yuyong Chen

**Affiliations:** 1 School of Materials Science and Engineering, Taiyuan University of Technology, Taiyuan 030024, China; zhshzh1984@163.com (S.Z.); zcj0408@163.com (C.Z.); 2 Shanxi Key Laboratory of Advanced Magnesium-based Materials, Taiyuan University of Technology, Taiyuan 030024, China; 3 School of Mechanical Engineering, Ningxia University, Ningxia 750021, China; slzhang1229@163.com; 4 Ningbo Branch of China Ordnance Academy, Ningbo 315000, China; yanfeichen@hotmail.com; 5 School of Mechanical Engineering, Taiyuan University of Technology, Taiyuan 030024, China; hanjianchao@tyut.edu.cn; 6 National Key Laboratory for Precision Hot Processing of Metals, Harbin Institute of Technology, Harbin 150001, China; yychen@hit.edu.cn

**Keywords:** intermetallics, rapid-solidification, melt extracted, Ti_5_Si_3_, divorced eutectic, TEM

## Abstract

The microstructure of melt extracted Ti-44Al-8Nb-0.2W-0.2B-1.5Si fiber were investigated. When the rotation speed increased from 2000 to 2600 r/min, the appearance of the wire was uniform with no Rayleigh-wave default. The structure was mainly composed of fine α_2_ (α) phase dendritic crystal and a second phase between dendrite arms and grain boundaries. The precipitated second phases were confirmed to be Ti_5_Si_3_ from the eutectic reaction L→Ti_5_Si_3_ + α and TiB. As the lower content of Si and higher cooling rate, a divorced eutectic microstructure was obtained. Segregation of Ti, Nb, B, Si, and Al occurred during rapid solidification.

## 1. Introduction

Due to the low density, high specific strength, elastic modulus, and oxidation resistance at high temperatures, γ-TiAl-based alloys have been considered as strong candidates for high-temperature structural applications in aerospace and automotive industries [1,2,3]. However, the poor hot workability and low room temperature ductility severely restrict their application [4,5]. Many efforts have been performed to enhance the mechanical properties, such as adding alloying elements [6,7], heat treatment [8], and thermomechanical treatment [9,10]. However, as the most effective method, thermomechanical treatment can only refine the grain size to the range of 5–10 μm and is always difficult to completely break down α_2_/γ lamellar structures [11,12].

Rapid solidification (RS) process could significantly refine grain size, produce metastable, and novel structures, increases solubility of alloying elements and reduce levels of segregation [13,14,15]. RS has been performed on binary TiAl alloys by different methods [16]. For RS, a single roller melt-spinning technique with circumferential velocities of 10–30 m/s has been utilized to produce TiAl ribbons [17,18]. However, the ribbon thickness, which affects cooling rate, is easily influenced by the melt temperature, and the mechanical properties of ribbon is limited by the defects of the surface [19,20]. Melt extraction is another melt spinning technique, which could prepare metallic fiber or ceramic fiber with diameters less than 30 μm, and a detailed description of this method is elsewhere [21]. Melt extraction not only allows access to relatively reactive metals, such as aluminum, titanium, zirconium, and magnesium, but also makes the fabrication of high temperature ceramics relatively easy [21,22]. Additionally, the cooling rate of melt extraction can reach about 106 K/s which is usually higher than other RS techniques [20,23].

Si element can enhance the creep resistance by forming the finer eutectoid Ti_5_Si_3_ phase, which has been well studied under conventional cast TiAl alloys [24]. However, the effect of RS on the solid solubility of Si and the formation of silicide in TiAl alloy has not been fully understood. Additional, the primary phase of TiAl alloy fabricated by single roller melt-spinning technique was β phase [18]. As the higher cooling rate of melt extraction than other RS technique, greater undercooling which strongly influences the primary phase and solidification path can be obtained.

The aim of this work was to investigate the microstructure characterization and prior phase of TiAl fiber under higher cooling rates fabricated by melt extraction. The formation of second phase, especially Ti_5_Si_3_ and its distribution were studied. In addition, the element segregation during rapid solidification was also investigated.

## 2. Experimental

Ti-44Al-8Nb-0.2W-0.2B-1.5Si alloy was fabricated by a Vacuum Arc Remelting furnace (120/200, Jinzheng Metallurgical Technology Corporation, Shenyang, China), with dimensions of φ 110 mm × 140 mm, and subsequently hot isostatic pressed (HIPed) at 1300 °C/130 MPa for 3 h under argon atmosphere. The raw materials were sponge titanium (99.9 wt %), pure Al (99.99 wt %), pure Si (99.9 wt %), Al-Nb (Nb: 50.9 wt %) master alloy, Al-W (W: wt %) master alloy, and B (99.7 wt %) crystals. The Ti-44Al-8Nb-0.2W-0.2B-1.5Si alloy ingots were cut into 10 mm in diameter and 10 mm in length by the electrical discharge machine. TiAl alloy fibers were prepared by the melt extraction technique using a copper wheel with diameter of 160 mm. The rotation speed of the copper wheel was 2000 and 2600 r/min, and the feed rate of the molten alloy is about 70 μm/s.

The morphology of fiber was characterized by scanning electron microscopy (SEM) in a TESCAN MIRA3 LMH field-emission scanning electron microscope (Tescan, Brno, Czech Republic). Microstructures were also observed via transmission electron microscopy (TEM) in a FEI Talos F200× field-emission environmental transmission electron microscope (FEI, Hillsboro‎, ‎OR, USA‎). Specimens for TEM were cross-sectioned by focused ion beam (FIB, HELIOS NanoLab 600i, FEI).

## 3. Results

**Figure 1:**
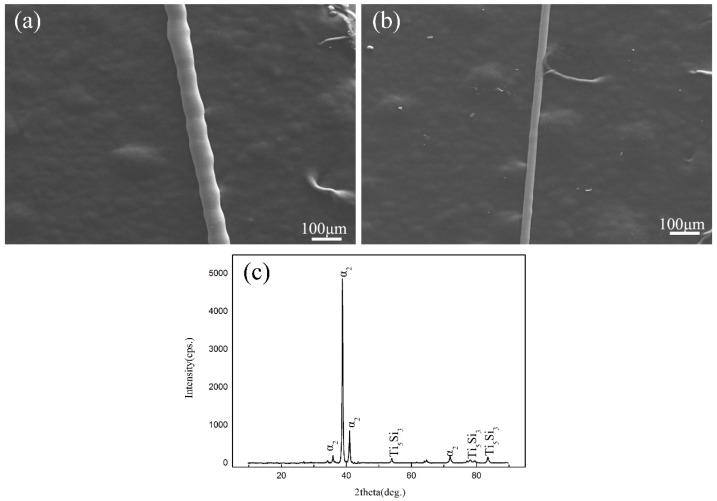
SEM micrograph of fibers with different rotation speeds and XRD results of fibers: (**a**) 2000 r/min; (**b**) 2600 r/min; (**c**) XRD results of fibers.

The different geometrical morphologies of fibers produced with different rotation speeds of the copper wheel are shown in Figure 1. The fiber fabricated with a rotation speed of 2000 r/min was of heterogeneous appearance which was the result of the instability of the extracted molten metal (Figure 1a). With the rotation speed increased to 2600 r/min, the appearance of the wire was uniform with no Rayleigh-wave default, shown in Figure 1b. Compared with higher rotate speed, as the decrease of rotate speed, the liquid metal cylinder which is under the effect of surface tension and its own weight is in its unstable state and forms a Rayleigh-wave default. Figure 1c is the XRD result of wire fabricated with rotation speed of 2600 r/min. It was revealed that the dominant phase of wire was α phase, with Ti_5_Si_3_ as one of the second phase. The microstructure subsequent studied was sampling from wires with a rotation speed of 2600 r/min.

**Figure 2:**
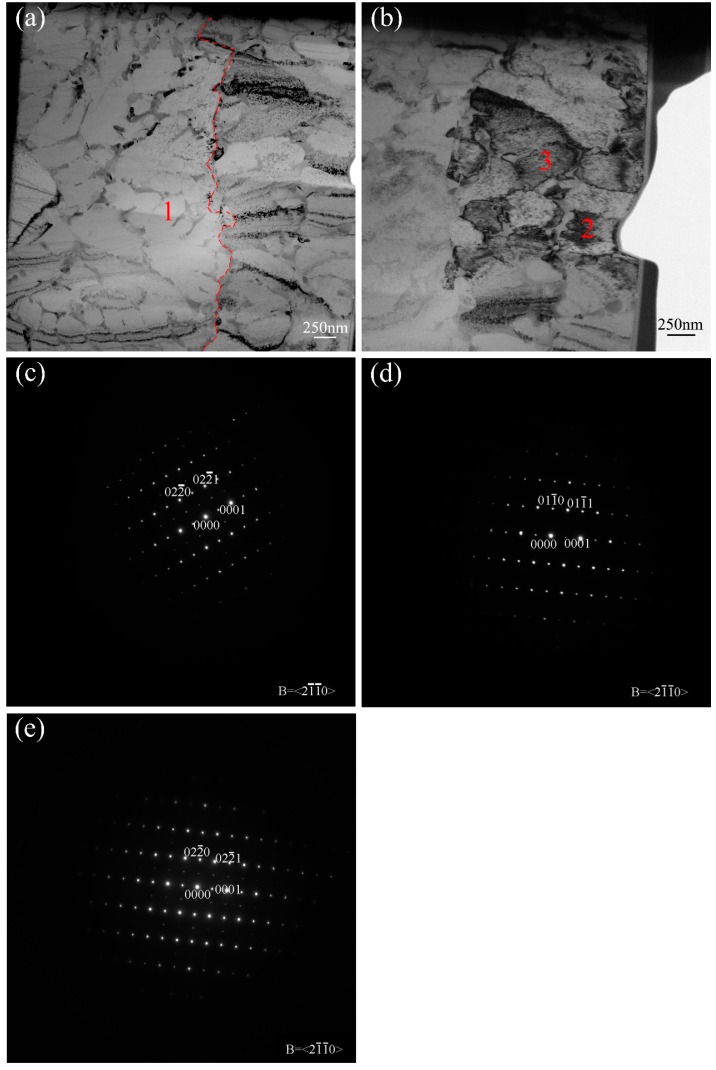
Cross-sectional TEM micrographs of the wires. (**a**,**b**) are the bright field images; (**c**–**e**) are the selected area diffraction patterns of regions in (a) and (b) marked by number 1, 2, and 3. Wheel contact surface is on the left of (a,b).

The microstructure of fibers was analyzed by TEM, Figure 2. As is shown in Figure 2a, there existed two different microstructural morphologies which had been divided by red dotted line. The structure on the left side was mainly composed of fine dendritic crystal and second phases located between the dendrite arms and at grain boundaries (Figure 2a), which is totally different from that of ribbons fabricated by RS technique [18]. The selected area diffraction (SAD) revealed that the dendrites were α_2_-Ti_3_Al (Figure 2), and the second phase were Ti_5_Si_3_ which were confirmed by HRTEM. The microstructure on the right side of Figure 2a away from the wheel contact surface of wire was a mixture structure of α_2_ and Ti_5_Si_3_, has no dentrite morphology, shown in the right side of Figure 2b. SAD illustrated that not all the α phase has ordered into α_2_ phase (Figure 2c,d). 

**Figure 3:**
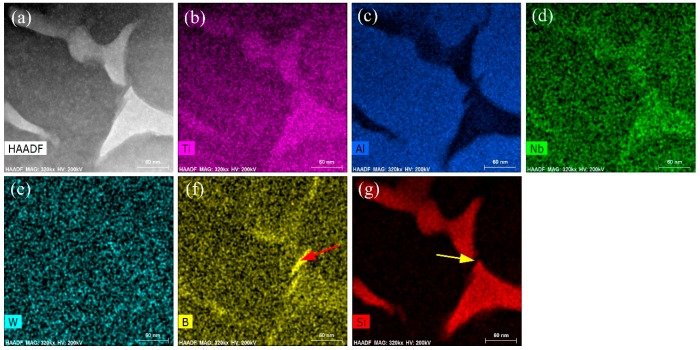
EDS chemical mapping of RS Ti-44Al-8Nb-0.2W-0.2B-1.5Si alloy: (**a**) The corresponding STEM-HADDF image; (**b**) Ti-Kα; (**c**) Al-Kα; (**d**) Nb-Kα; (**e**) W-Kα; (**f**) B-Kα; and (**g**) Si-Kα.

In order to understand the distribution of elements, EDS mapping was carried out of the RS Ti-44Al-8Nb-0.2W-0.2B-1.5Si alloy, Figure 3 EDS results revealed that W element was homogeneous in distribution. As a β phase stabilizing element, W segregated in β phase at grain boundaries during conventional solidification. Compared with conventional solidification, the RS technique has improved the solubility of W element in α_2_ phase. On the contrary, the elements Ti, Al, Nb, B, and Si were significantly segregated. The Ti, Nb, B, and Si elements were segregated to the second phase between the dendrite arms and along grain boundaries. The second phase in the dendritic arms and along grain boundaries was enriched with Ti and Si, but without Al, and Si element was only interdendritic, but Al element enriched the matrix phase (α phase or α_2_ phase). Si and B segregated to different phase, Figure 3f,g.

**Figure 4:**
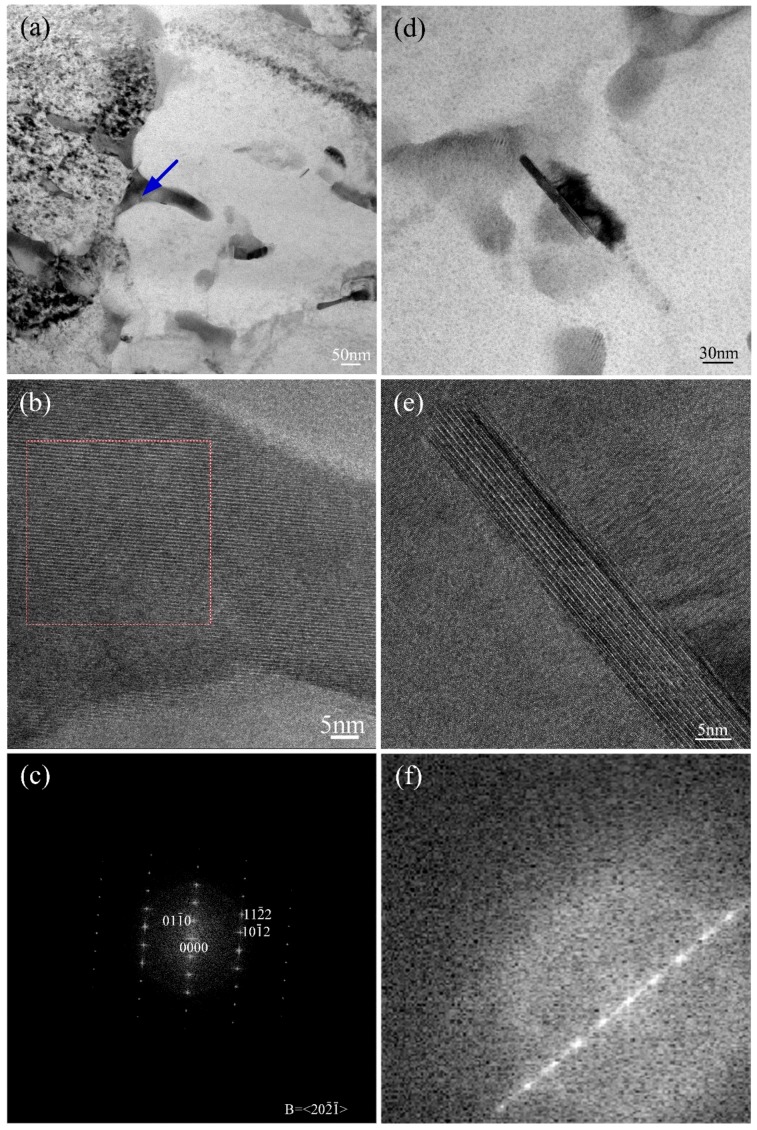
TEM micrographs of second phases: (**a**,**d**) are bright field images; (**b**,**c**) are the HRTEM and FFT of the region marked by blue arrow in (a); (**e**,**f**) are the HRTEM and FFT of the rod-like phase in (b).

The further study of the second phase was carried out by TEM, as shown in Figure 4. Figure 4a shows the morphology and the distribution of second phase. HRTEM was performed to study the region marked by the blue arrow in Figure 4a, and the FFT graph of this area is revealed by Figure 4c. As is shown by Figure 4a, the length of the phase indicated by arrow is no more than 200 nm. The precipitated second phase was confirmed as Ti_5_Si_3_ by HRTEM and the corresponding FFT result. In order to further understand the morphology of the B enriched phase, Figure 4d, a bright field image was carried out. The B enriched phase with a rod-like shape always precipitated accompanying Ti_5_Si_3_ along grain boundaries. As the small size of the B-rich phase, the diffraction spot in the FFT image could not be used to analyses the crystal structure, shown in Figure 4e,f. However, TiB is the only boride produced in TiAl based alloy when B element is lower [22].

## 4. Discussion

According to the analysis above, the main phase of RSTi-44Al-8Nb-0.2W-0.2B-1.5Si fiber was α_2_ (α), was totally different from the conventional casting which is typically β phase solidification alloy with a β phase as primary phase [25]. When the rotation speed reached 2600 r/min, the high undercooling resulting from the high cooling rate of RS is beneficial to the formation of primary α phase [18,26]. Compared to Ti-48Al-2Cr alloy strip obtained by single roller melt-spinning technique, there was no β phase in the structure ofTi-44Al-8Nb-0.2W-0.2B-1.5Si alloy wires becuase the cooling rate of melt extraction technique is higher than that of single roller melt-spinning technique [18]. Although the solid solubility of Si in α phase could reach up to 15 at %, the presence of Nb element, which is a strong silicide former reduced the solubility for Si in the α_2_-Ti_3_Al phase [24,27]. Si was ejected into the melt during rapid solidification, and formed silicide at the solid-liquid interface between α phase and liquid. As revealed by Figure 2b–d, not all the high temperature α phase has transformed into its ordered state α_2_ phase, and there still exists α phase which is probably the result of rapid cooling rate. Meanwhile, TiB has also been detected in the microstructure ofTi-44Al-8Nb-0.2W-0.2B-1.5Si wires. 

Ti_5_Si_3_ distributed at the dendrite arms is the primary second phase in the structure of Ti-44Al-8Nb-0.2W-0.2B-1.5Si wires. As illustrated by Sun, primary large isolated Ti_5_Si_3_, eutectic morphology Ti_5_Si_3_ whisker (L→Ti_5_Si_3_ + α) and eutectoid Ti_5_Si_3_ (α→Ti_5_Si_3_ + γ) had been found in TiAl alloy with different Si containing [28]. With the decrease of temperature and the changes of melt constituent after the formation of primary α phase (α_p_) of RS TiAl alloy wires, a eutectic transformation of L→Ti_5_Si_3_ + α took place (α_e_). For the Si content is merely 1.5 at %, a large volume fraction of α_p_ is acquired before the eutectic transformation. That is to say, hypoeutectic microstructure could obtain. As is shown in Figure 2, the high cooling rate changes the morphology of hypoeutectic microstructure to divorced eutectic which has the morphology that the eutectic Ti_5_Si_3_ was separated along α phase grain boundaries rather than formed eutecticum. Divorced eutectic could be obtained under the condition of alloy composition is far away from eutectic point or non-equilibrium solidification (e.g., higher cooling rate) [29,30]. The divorced eutectic has no longer had the alternate characteristic of eutectic structure and has the two phase separated with each other. The α_e_ phase nucleated on the interface of α_p_ phase and then grown up, and the eutectic Ti_5_Si_3_ phase was push out to the grain boundary of the α_p_ phase. Based on the Ti-Al-B ternary phase diagram, alloy with the composition of Ti-44Al-0.2B has the final microstructure of α_2_ + γ + TiB2, and TiB2 was the stable boride [31]. However, the phase composition of RS Ti-44Al-8Nb-0.2W-0.2B-1.5Si alloy was α (α_2_) and TiB which is the stabilized boride. Two reasons could be in consider, on the one hand, RS has caused the phase field which the solidification microstructure located in shifting left towards the lower aluminum content on the equilibrium phase diagram. That is to say, the microstructure of Ti-44Al-8Nb-0.2W-0.2B-1.5Si changed from α_2_ + γ + TiB2 to α (α_2_) + TiB. On the other hand, as β stabilizing elements, Nb and W strongly influenced the solidification path, and then TiB was the stable boride in the microstructure of RS Ti-44Al-8Nb-0.2W-0.2B-1.5Si alloy. As reported by Hu, the formation of TiB at solid-liquid interface is beneficial to the formation of alpha phase [32,33]. However, in the microstructure of this RSTi-44Al-8Nb-0.2W-0.2B-1.5Si wire, TiB is not as nucleus of α phase. It is maybe the reason why TiB could not be as the nucleus of α phase is that the volume fraction of α_p_ phase is large and then the later α phase forms in the remnant melt at the position of the interface between α_p_ phase and remnant melt rather than at the position of TiB. This nucleation mode has lower interfacial energy and distortion energy than that of the nucleation with TiB. 

## 5. Conclusions

RS Ti-44Al-8Nb-0.2W-0.2B-1.5Si wire was fabricated by melt extraction technique. The appearance of the wire was fine and uniform when the rotation speed increased to 2600 r/min. TEM analysis revealed that the structure is mainly composed of fine dendritic crystal and second phase (Ti_5_Si_3_ and TiB) between dendrite arms and at grain boundaries. As the result of high cooling rate, α phase nucleated as primary phase, and some α phase was remain rather than ordered into α_2_ phase. The formation of Ti_5_Si_3_ is the result of the eutectic transformation of L→Ti_5_Si_3_ + α. However, for the hypoeutectic microstructure and higher cooling rate, a divorced eutectic microstructure was obtained. The solubility of W element in α_2_ phase was improved by RS, but the segregation of Ti, Nb, B, Si, and Al was not obviously influenced by the cooling rate. TiB is the only boride containing in the microstructure of RS Ti-44Al-8Nb-0.2W-0.2B-1.5Si alloy.

## References

[1] Zhang T., Wu Z., Hu R., Zhang F., Kou H., Li J. (2016). Influence of nitrogen on the microstructure and solidification behavior of high Nb containing TiAl alloys. Mater. Des..

[2] Shen Z.Z., Lin J.P., Liang Y.F., Zhang L.Q., Shang S.L., Liu Z.K. (2015). A novel hot pack rolling of high Nb-TiAl sheet from cast ingot. Intermetallics.

[3] Han J., Xiao S., Tian J., Chen Y., Xu L., Wang X., Jia Y., Rahoma H.K.S., Du Z., Cao S. (2015). Microstructure characterization, mechanical properties and toughening mechanism of TiB2-containing conventional cast TiAl-based alloy. Mater. Sci. Eng. A.

[4] Rashkova B., Spiradek-Hahn K., Brabetz M., Zhang Z.L., Schoberl T., Clemens H., Mayer S. (2015). Microstructural evolution and grain refinement in an intermetallic titanium aluminide alloy with a high molybdenum content. Int. J. Mater. Res..

[5] Klein T., Schachermayer M., Mendez-Martin F., Schöberl T., Rashkova B., Clemens H., Mayer S. (2015). Carbon distribution in multi-phase γ-TiAl based alloys and its influence on mechanical properties and phase formation. Acta Mater..

[6] Imayev R.M., Imayev V.M., Oehring M., Appel F. (2007). Alloy design concepts for refined gamma titanium aluminide based alloys. Intermetallics.

[7] Kartavykh A.V., Asnis E.A., Piskun N.V., Statkevich I.I., Gorshenkov M.V., Tcherdyntsev V.V. (2014). Lanthanum hexaboride as advanced structural refiner/getter in TiAl-based refractory intermetallics. J. Alloys Compd..

[8] Schwaighofer E., Clemens H., Mayer S., Lindemann J., Klose J., Smarsly W., Güther V. (2014). Microstructural design and mechanical properties of a cast and heat-treated intermetallic multi-phase γ-TiAl based alloy. Intermetallics.

[9] Schwaighofer E., Clemens H., Lindemann J., Stark A., Mayer S. (2014). Hot-working behavior of an advanced intermetallic multi-phase γ-TiAl based alloy. Mater. Sci. Eng. A.

[10] Cui N., Kong F., Wang X., Chen Y., Zhou H. (2016). Microstructural evolution, hot workability, and mechanical properties of Ti-43Al-2Cr-2Mn-0.2Y alloy. Mater. Des..

[11] Zhang S.Z., Kong F.T., Chen Y.Y., Liu Z.Y., Lin J.P. (2012). Phase transformation and microstructure evolution of differently processed Ti-45Al-9Nb-Y alloy. Intermetallics.

[12] Dong S., Chen R., Guo J., Ding H., Su Y., Fu H. (2015). Deformation behavior and microstructural evolution of directionally solidified TiAlNb-based alloy during thermo-compression at 1373–1573 K. Mater. Des..

[13] Huang S.-C., Hall E.L. (1991). Characterization of the effect of vanadium additions to TiAl base alloys. Acta Metall. Mater..

[14] Shao G., Grosdidier T., Tsakiropoulos P. (1994). The metastable disordered γ (TiAl) phase and its ordering process in a rapidly solidified equiatomic TiAl alloy with vanadium addition. Scr. Metall. Mater..

[15] Karaköse E., Keskin M. (2009). Effect of solidification rate on the microstructure and microhardness of a melt-spun Al-8Si-1Sb alloy. J. Alloys Compd..

[16] Liu Y.C., Yang G.C., Guo X.F., Huang J., Zhou Y.H. (2001). Coupled growth behavior in the rapidly solidified Ti-Al peritectic alloys. J. Cryst. Growth.

[17] Liu Z.G., Chai L.H., Chen Y.Y., Kong F.T., Davies H.A., Figueroa I.A. (2011). Microstructure evolution in rapidly solidified Y added TiAl ribbons. Intermetallics.

[18] Zhu D., Dong D., Ni C., Zhang D., Zhou Z., Wang H., Wei Z. (2015). Effect of wheel speed on the microstructure and nanohardness of rapidly solidified Ti-48Al-2Cr alloy. Mater. Charact..

[19] Phan M.-H., Peng H.-X. (2008). Giant magnetoimpedance materials: Fundamentals and applications. Prog. Mater. Sci..

[20] Ström-Olsen J. (1994). Fine fibres by melt extraction. Mater. Sci. Eng. A.

[21] Wang H., Xing D., Wang X., Sun J. (2011). Fabrication and characterization of melt-extracted Co-based amorphous wires. Metall. Mater. Trans. A.

[22] Allahverdi M., Drew R.A.L., Strom-Olsen J.O. (1996). Melt-extracted oxide ceramic fibers—The fundamentals. J. Mater. Sci..

[23] Ma X.Z., Shen J., Jia J. (2001). Study on rare earth-containing phases in TiAl based alloys prepared by non-equilibrium solidification processing. J. Rare Earth.

[24] Klein T., Rashkova B., Holec D., Clemens H., Mayer S. (2016). Silicon distribution and silicide precipitation during annealing in an advanced multi-phase γ-TiAl based alloy. Acta Mater..

[25] Yang G., Kou H., Yang J., Li J., Fu H. (2016). Microstructure control of Ti45Al8.5Nb(W, B, Y) alloy during the solidification process. Acta Mater..

[26] Huang S.-C., Hall E.L. (1991). The effects of Cr additions to binary TiAl-base alloys. Metall. Trans. A.

[27] Dezellus O., Gardiola B., Andrieux J., Lomello-Tafin M., Viala J.C. (2014). On the liquid/solid phase equilibria in the Al-rich corner of the Al-Si-Ti ternary system. J. Ph. Equilib. Diffus..

[28] Sun F.-S., Froes F.H. (2003). Solidification behavior of Ti_5_Si_3_ whiskers in TiAl alloys. Mater. Sci. Eng. A.

[29] Yan N., Geng D.L., Hong Z.Y., Wei B. (2014). Ultrasonic levitation processing and rapid eutectic solidification of liquid Al-Ge alloys. J. Alloys Compd..

[30] Dahle A.K., Lee Y.C., Nave M.D., Schaffer P.L., St John D.H. (2001). Development of the as-cast microstructure in magnesium-aluminium alloys. J. Light Met..

[31] Witusiewicz V.T., Bondar A.A., Hecht U., Zollinger J., Artyukh L.V., Velikanova T.Y. (2009). The Al-B-Nb-Ti system: V. Thermodynamic description of the ternary system Al-B-Ti. J. Alloys Compd..

[32] Hu D., Yang C., Huang A., Dixon M., Hecht U. (2012). Solidification and grain refinement in Ti45Al2Mn2Nb1B. Intermetallics.

[33] Hu D., Yang C., Huang A., Dixon M., Hecht U. (2012). Grain refinement in beta-solidifying Ti44Al8Nb1B. Intermetallics.

